# Best of 2023 in psychosomatic medicine: the contribution to the rest of psychiatry

**DOI:** 10.1192/j.eurpsy.2024.32

**Published:** 2024-08-27

**Authors:** S. Ferrari

**Affiliations:** University of Modena & Reggio Emilia, Modena, Italy

## Abstract

**Abstract:**

The field of psychosomatics has experienced many waves of “celebrity” since its origin. Its historical origin is impossible to precisely locate in time, one may argue that medicine since its very beginning has been psychosomatic in nature. In very recent times, many clinicians and researchers even from different backgrounds than psychosomatic medicine or psychiatry have expressed disappointment and worry about the excessive fragmentation of medical sciences, providing evidence in support and advocating towards the so-called holistic approach and integrated care. The old lesson of psychosomatic medicine, then, appears more contemporary than ever. This is also because it has been able to stay coherent but at the same time integrate the enormous progresses in the understanding of physiology and pathophysiology that medical sciences have witnessed in the last decades.

The presentation will focus on the most striking scientific production of 2023 in the field of psychosomatics, to show the contributions in its three souls of research, training and clinical activities and to outline the stimulating though sometimes difficult dialogue between this area of behavioural sciences and the rest of psychiatry.

**Disclosure of Interest:**

None Declared

